# ATP Synthase C-Subunit-Deficient Mitochondria Have a Small Cyclosporine A-Sensitive Channel, but Lack the Permeability Transition Pore

**DOI:** 10.1016/j.celrep.2018.12.033

**Published:** 2019-01-02

**Authors:** Maria A. Neginskaya, Maria E. Solesio, Elena V. Berezhnaya, Giuseppe F. Amodeo, Nelli Mnatsakanyan, Elizabeth A. Jonas, Evgeny V. Pavlov

**Affiliations:** 1 College of Dentistry, Department of Basic Sciences, New York University, New York, NY 10010, USA; 2 Laboratory of Molecular Neurobiology, Sothern Federal University, Academy of Biology and Biotechnology, 344090 Rostov-on-Don, Russia; 3 Section of Endocrinology, Department of Internal Medicine, Yale University, New Haven, CT 06511, USA; 4 Lead Contact

## Abstract

Permeability transition (PT) is an increase in mitochondrial inner membrane permeability that can lead to a disruption of mitochondrial function and cell death. PT is responsible for tissue damage in stroke and myocardial infarction. It is caused by the opening of a large conductance (~1.5 nS) channel, the mitochondrial PT pore (mPTP). We directly tested the role of the c-subunit of ATP synthase in mPTP formation by measuring channel activity in c-subunit knockout mitochondria. We found that the classic mPTP conductance was lacking in c-subunit knockout mitochondria, but channels sensitive to the PT inhibitor cyclosporine A could be recorded. These channels had a significantly lower conductance compared with the cyclosporine A-sensitive channels detected in parental cells and were sensitive to the ATP/ADP translocase inhibitor bongkrekic acid. We propose that, in the absence of the c-subunit, mPTP cannot be formed, and a distinct cyclosporine A-sensitive low-conductance channel emerges.

## In Brief

Neginskaya et al. report that c-subunit-deficient mitochondria contain a CSA-sensitive channel. This channel is much smaller compared with the wild-type permeability transition pore and is sensitive to inhibitors of adenine nucleotide translocase. This work highlights the importance of the c-subunit in forming the permeability transition pore.

## Graphical Abstract

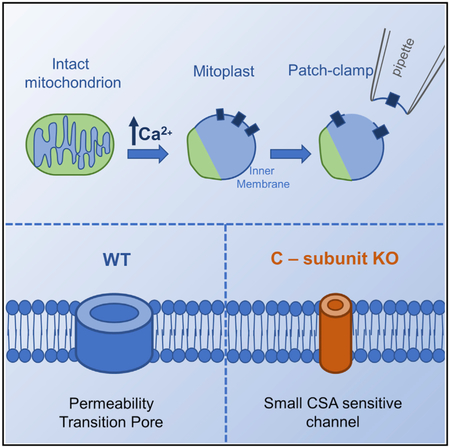

## INTRODUCTION

Mitochondrial permeability transition (PT) is the phenomenon of a dramatic increase in the permeability of the mitochondrial inner membrane. PT is triggered by excessive accumulation of calcium inside energized mitochondria. Activation of PT leads to mitochondrial membrane depolarization and disruption of ATP production. PT is believed to be the major cause of cell death during acute stress in conditions such as stroke and heart attack (Bernardi et al., 2006). It is established that PT is caused by the opening of a large channel (mitochondrial PT pore, or mPTP) in the inner membrane. The key characteristics of this pore are conductance of approximately 1.5 nS and sensitivity to cyclosporine A (CSA), a known inhibitor of PT in intact mitochondria and cells (Kinnally et al., 1989, 1992, 1996; Szabò and Zoratti, 1991, 1992). Molecular composition of the ion conducting pore of the mPTP channel is not well understood and is the subject of intensive investigation. Over the past few years, several reports have suggested that the ion-conducting part of mPTP might be formed by direct participation of the ATP synthase (Alavian et al., 2014; Bonoraet al., 2013; Elustondo et al., 2016; Giorgio et al., 2013). It has been proposed that the pore is directly formed by the c-subunit ring (Alavian et al., 2014), oligomers of the c-subunit of the ATP synthase (Bonora et al., 2017; Jonas et al., 2015), by assembly of c-subunit/polyphosphate/polyhydroxybutyrate complex (Elustondo et al., 2016; Pavlov et al., 2005), or by a putative channel-forming structure localized in between two monomers of this enzyme (Bernardi et al., 2015). The link between c-subunit channel and mPTP channel has been recently questioned by a study that used cultured HAP1-A12 cells that demonstrated that mitochondria lacking c-subunit can still undergo calcium induced and CSA-sensitive membrane depolarization, calcium and calcein release (He et al., 2017b). Here, we use c-subunit knockout mitochondria from HAP1-A12 cells to investigate the involvement of the c-subunit in mPTP. To do this, we use an electrophysiological approach to directly measure the channel activity of c-subunit-deficient mitochondria under conditions of PT. We find that, similar to wild-type, c-subunit knockout mitochondria contain channel activity sensitive to CSA (Halestrap and Davidson, 1990). However, the recorded channel had significantly lower conductance compared to the mPTP channel found in wild-type mitochondria. We propose that in the absence of the c-subunit, the large conductance mPTP channel cannot form, but opening of other CSA-sensitive channels may contribute to depolarization of the inner mitochondrial membrane.

## RESULTS

### mPTP Channel Activity Is Evoked by Calcium in Wild-Type Mitochondria Isolated from Wild-Type HAP1 Cells Containing the C-Subunit

**Figure 1.**
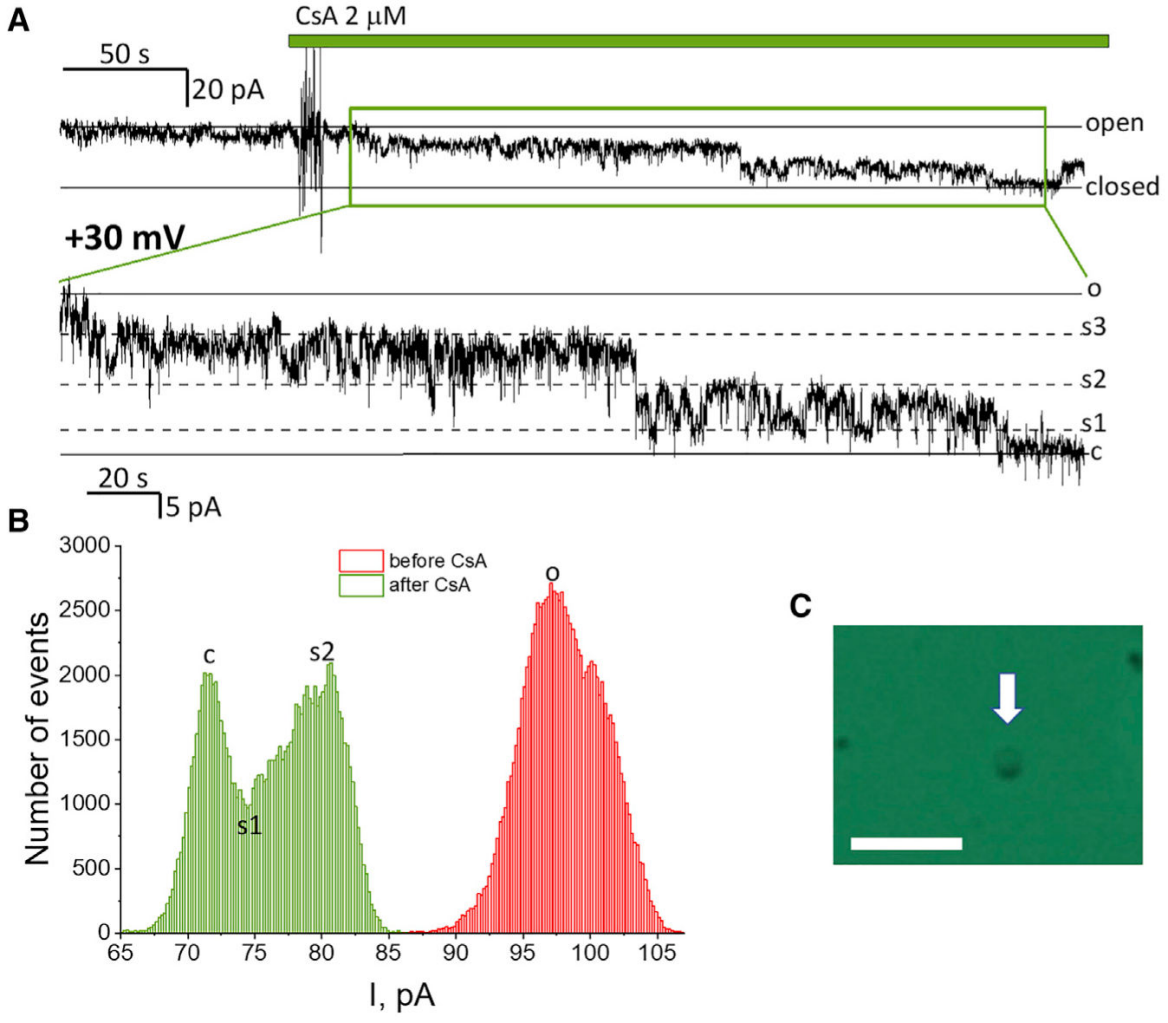
Channel Activity of the Permeability Transition Pore of Wild-Type HAP1 Cells Containing C-Subunit (A) Representative single channel current recording of n = 10 recordings. Note progressive transition of the channel form open to sub-conductance to fully closed state following addition of CSA. (B) All-points histogram of ionic current corresponding to the recording shown in (A). (C) Phase-contrast image of the mitoplast and patch-clamp pipette. Note the dark “cap” on the top of mitoplast formed by the remains of the mitochondrial outer membrane. Scale bar, 5 μm.

To study channel activity associated with PT, we used mitochondria isolated from cultured HAP1 cells (He et al., 2017b). Following isolation mitochondria were placed into a solution containing 150 mM KCl at pH = 7.4. Mitochondria were energized by the addition of succinate (4 mM) in the presence of rotenone (2 μM). Addition of 2 mM of CaCl_2_ resulted in swelling of mitochondria and rupture of the mitochondrial outer membrane that was evaluated by light microscopy in the phase contrast configuration. Typical mitoplasts form a spherical shape with one or two dark spots on the side (Figure 1C), which constitute the remaining fragments of the outer membrane. Ion channel activity was studied by direct patch-clamping of the mitoplasts’ membrane in excised patch configuration.

**Figure 2.**
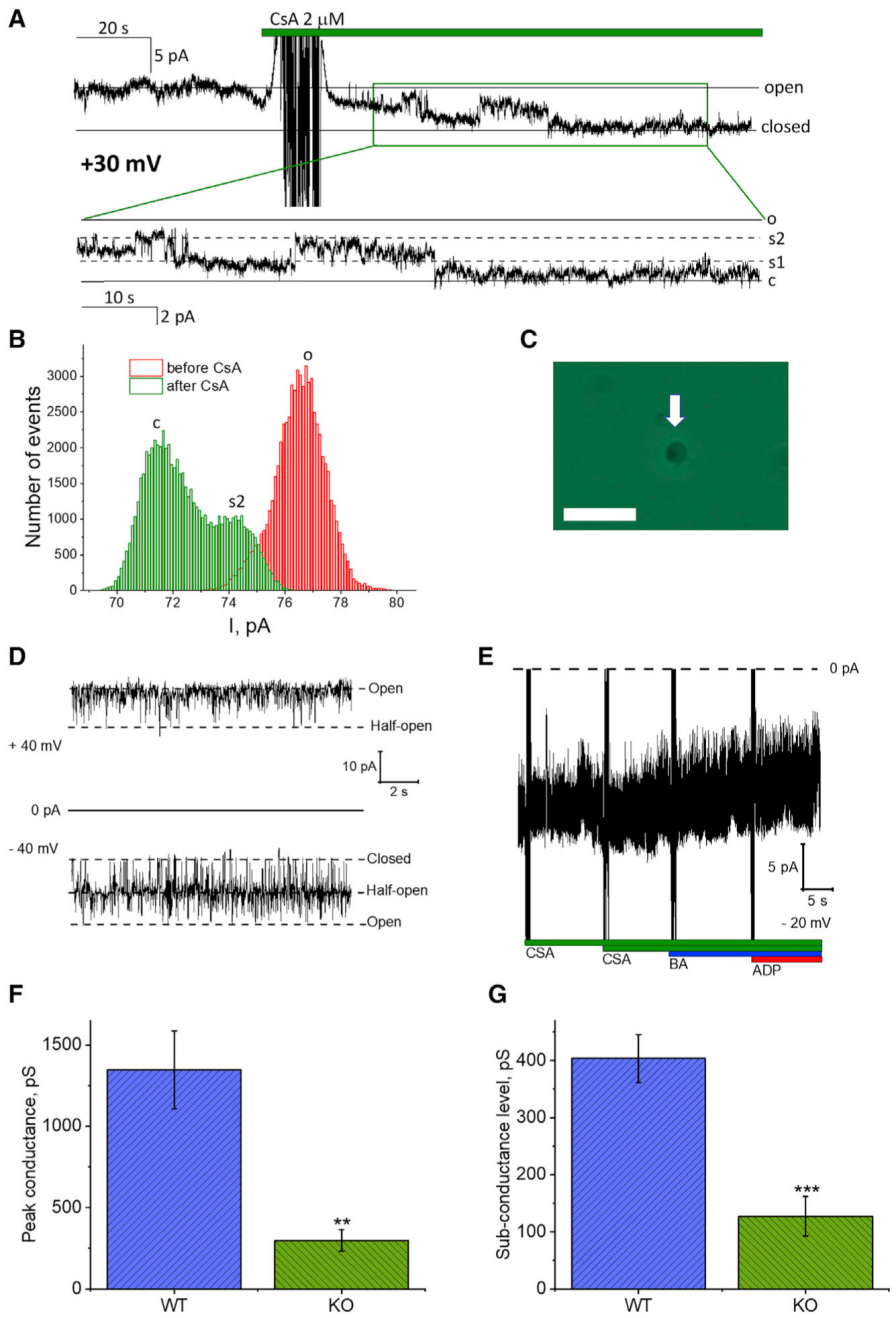
Activity of the CSA-Sensitive Channel of C-Subunit Knockout Cells (A) Representative single channel current recording of n = 10 recordings. Note progressive transition of the channel form open to sub-conductance to fully closed state following addition of CSA. (B) All-points histogram of ionic current corresponding to the recording shown in (A). (C) Phase-contrast image of the mitoplast from c-subunit knockout cells. Scale bar, 10 μm. (D) Typical recording of the TIM channel in the c-subunit knockout (KO) cells (see also Figure S1). (E) Lack of CSA, BA, and ADP sensitivity of the TIM channel; condensed trace of the currents shown in (D). (F and G) Peak conductance (F) and sub-conductance (G) values, respectively, of wild-type cells containing c-subunit and CSA-sensitive channels from c-subunit KO cells. Current values were calculated based on single channel recordings similar to the one shown in Figures 1A and 2A. n = 10 for wild-type (WT) and n = 10 for KO. Data are represented as mean ± SEM. **p < 0.005; ***p < 0.001.

Figure 1A shows a representative recording of the channel activity in a mitoplast that was pretreated with calcium. Prior to the addition of the mPTP inhibitor, CSA, the channel remained in a predominantly open state with brief closures to a sub-conductance state. Channel activity was detected in 29% of such patches (16 of 55). Addition of the mPTP inhibitor CSA (2 μM) caused inhibition of the channel (Figures 1A and 1B) with stepwise transition from the fully open to the fully closed state. In our experiments we considered the difference in conductance before and after addition of CSA as the measure of the conductance of the fully open mPTP. These data analysis criteria allowed to focus on CSA-sensitive mPTP and excluded other types of ion conductance that can be detected in the mitochondrial native membranes. The representative CSA-sensitive trace in Figure 1A shows the typical current recording that was used for data analysis. Figure 1B shows the all-points histogram of the single channel recording that allows a more accurate determination of the values of the ionic currents. The average channel size of the CSA-sensitive channels, as measured from the fully open to the fully closed (calculated based on the minimal ionic current measured in the presence of CSA) state was 1.3 ± 0.2 nS (n = 10 independent experiments) (Figures 1 and 2F). The amplitude of the predominant transition between substates (see, for example, the sub-states labeled “S1” and “S2” in the expanded trace on Figure 1A) of these channels was 400 ± 40 pS (n = 10 independent experiments) (Figures 1 and 2G). Overall, we conclude that under conditions of calcium activation mitochondria from wild-type HAP1 cells contain CSA-sensitive channel activity with conductance and kinetics characteristic of the mPTP channel described in earlier studies (Kinnally etal., 1989; Szabò and Zor-atti, 1991).

### CSA-Sensitive Channel Activity of C-Subunit Knockout Mitochondria Has Low Conductance

Next, we investigated the ion channel activity of the mutant mitochondria from HAP1-A12 cells lacking the c-subunit of the ATP synthase. Patch clamp recordings were performed under conditions identical to those of mitochondria from wild-type HAP1 cells. Criteria for data analysis were chosen in the same way as for HAP1 cells. Our protocol produced mitoplasts of similar appearance (as seen by phase contrast bright field microscope) compared to wild-type mitoplasts (Figure 2C). Channel detection frequency was not dramatically altered compared to that of wild-type mitoplasts, with 43% of patches demonstrating channel activity (20 patches of 46). Similar to the wild-type, in the absence of CSA channels remained mostly in a fully open state with brief closures to sub-conductance states (Figure 2A). Within the voltage range from −30 to +30 mV that were used in our experiments the channel activity did not demonstrate significant voltage dependence. Following channel detection, we tested whether these channels were sensitive to CSA. Addition of 2 μM CSA led to channel inhibition in 10 experiments. In these experiments we observed a progressive transition of the channels to lower conductance sub-states and eventual closure (Figure 2A). The average conductance of the fully open state was 300 ± 70 pS (n = 10 independent experiments) while the amplitude to the predominant transition between sub-conductance states was 130 ± 30 pS (n = 10 independent experiments). Both the average maximal conductance and sub-states conductance of the channels seen in c-subunit knockout (KO) cells were significantly lower compared to the CSA-sensitive channel activity recorded from the mitochondria of wild-type cells (Figures 2F and 2G).

In addition to CSA-sensitive channels, we detected channel activity that was not sensitive to CSA. The behavior of these channels was characteristic of the previously reported activity of the translocator of the inner membrane (TIM) (Figures 2D and S1). Note presence of the typical half-conductance substate, much more frequent gating and tendency to close at negative voltages compared to positive voltages. Taking into account that the TIM channel was not sensitive to CSA (Figure 2E), and that earlier studies have suggested TIM is not part of Mptp (Grigoriev et al., 2004), we conclude that the TIM channel recorded in our experiments is not responsible for activation of the CSA-sensitive PT.

### CSA-Sensitive Channel in C-Subunit KO Mitochondria Is Also Sensitive to the ANT Inhibitor Bongkrekic Acid

**Figure 3.**
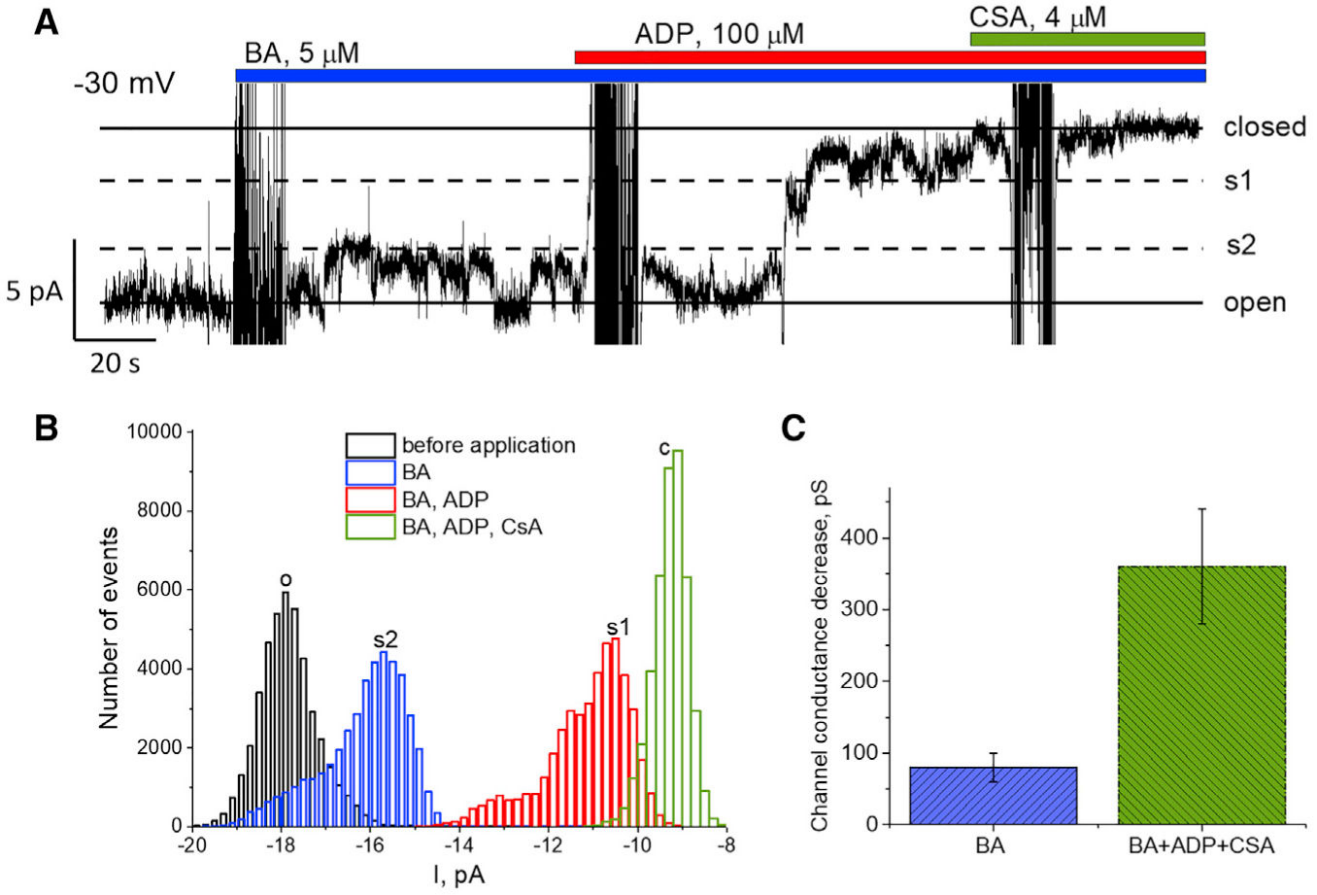
Sensitivity of the Channel from C-Subunit KO Mitochondria to the Ligands of the Adenine Nucleotide Translocator (A) Representative single-channel ion current recording in the presence of different blockers. Note progressive decrease of channel conductance. (B) All-points histogram corresponding to the ion current shown in (A). This behavior was observed in 4 of 15 experiments. (C) Decrease in channel conductance in the presence of ANT and PTP inhibitors (n = 3).

Our experimental results indicate that deletion of the c-subunit leads to disappearance of the1.5 nS, CSA-sensitive large conductance channel that has been accepted previously as that of mPTP (Kinnally et al., 1996; Zoratti and Szabò, 1995). Along with the lack of this large conductance channel, we found an increased detection frequency of a of 300 pS channel that was also sensitive to CSA. While to our knowledge, the presence of such CSA-sensitive channel activity in native membranes has never been reported, previous studies indicate that both purified and recombinant adenine nucleotide translocator (ANT) of the mitochondrial inner membrane can demonstrate CSA-sensitive channel activity (Brustovetsky and Klingenberg, 1996) in artificial lipid membranes when experiments were performed in the presence of Cyclophilin D (CypD), the known endogenous target of CSA. Interestingly in these studies recombinant ANT when reconstituted into artificial lipid bilayers demonstrated a peak conductance in the range of 300 pS that is comparable to the conductance of the channel seen in our experiments with c-subunit KO mitoplasts. Thus, we hypothesized that in the absence of c-subunit, the CSA dependent behavior recorded in mitoplasts might be caused by activation of ANT channel. To test this, we probed the channels recorded in c-subunit KO mitoplasts with antagonists of ANT. In this experimental protocol addition of bongkrekic acid (BA) is expected to cause transition of ANT to sub-conductance states which can be nearly completely blocked by ADP (Brustovetsky and Klingenberg, 1996). We found that in c-subunit KO mitoplasts, addition of 5 μM of the ANT blocker BA caused channel transition to a lower conductance sub-state (Figures 3A and 3B). It should be noted that the trace in Figure 3A was recorded at negative membrane potential and thus the all-points histogram in Figure 3B has negative current values. Addition of 100 μM ADP in the presence of BA led to further inhibition of the channel. Finally, addition of 4 μM CSA led to complete inhibition of the channel (Figures 3A and 3B). This behavior is characteristic of the channel activity of previously reported reconstituted purified ANT. This type of behavior was detected in 4 of 15 of our experiments. The overall peak conductance of the patches with clear single channel activity sensitive to sequentially added BA, ADP, and CSA was 360 ± 80 pS (n = 3), with BA alone blocking 80 ± 20 pS (n = 3) (Figure 3C). In another four experiments, channel activity was not sensitive to any of these pharmacological reagents and demonstrated channel behavior characteristic of TIM. In the remainder of the experiments either smaller channel activity or no channel activity was detected, and these recordings did not contain activity sensitive to any of the aforementioned inhibitors. Importantly, all the recordings that were not sensitive to BA and ADP were also insensitive to CSA. Altogether these experiments suggest that in c-subunit KO mitochondria, CSA-sensitive channel activity is consistent with that of ANT. We should note that in our experimental approach it was not possible to use a similar type of analysis to investigate the regulation of the mPTP channel in wild-type mitochondria because both ANT and ATP-synthase channels are sensitive to CSA and adenine nucleotides, making data interpretation ambiguous. It is difficult to differentiate between the two proteins based only on the response to inhibitors.

### Regulation of Calcium-Induced Mitochondrial Membrane Depolarization in Intact C-Subunit KO Cells

**Figure 4.**
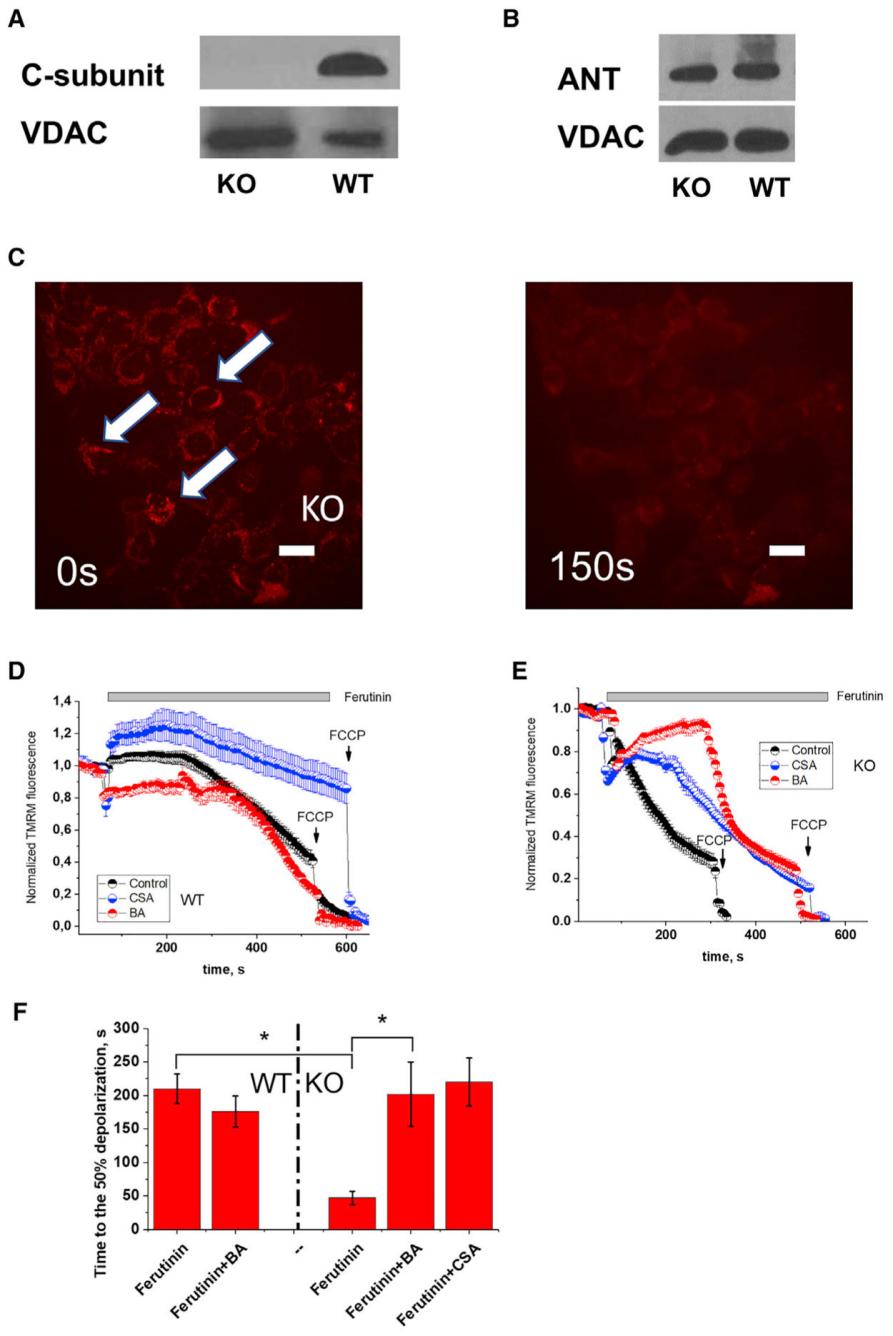
Calcium-Induced Mitochondrial Membrane Depolarization Occurs in Intact C-Subunit KO Cells (A) Western blot of the levels of c-subunit in WT HAP1 cells and KO HAP1 – A12 cells. Note absence of detectable signal in the KO cells. (B) Western blot detection of ANT in both WT and c-subunit KO cells. (C) Representative images of TMRM fluorescence before and after addition of the Ca^2+^ ionophore ferutinin (10 μM). Scale bar, 20 μm. (D) Time dependence of mitochondrial membrane depolarization in HAP1 cells containing c-subunit following addition of ferutinin (10 μM) in control and in the presence of CSA (4 μM) and BA (5 μM) (traces represent mean ± SEM of n = 10 ROI). (E) Time dependence of mitochondrial membrane depolarization in c-subunit KO cells following addition of ferutinin. Note significant delay in the presence of 5 μM of BA (traces represent mean ± SEM from n = 10 ROI). (F) Left: time to onset of depolarization in WT cells was not different in the absence and presence of 5 μM of BA (n = 5 for control and n = 3 for BA) expressed as the time to 50% depolarization. Right: time to onset of depolarization in c-subunit KO cells in the absence and presence of inhibitors expressed as the time to 50% depolarization (n = 6, control; n = 4, BA; n = 3 CSA). Data are represented as mean ± SEM. *p < 0.05.

Next, we tested whether BA sensitivity can be detected at the level of intact cells. Using immunoblotting, we confirmed that, as expected, these cells lack c-subunit and express ANT (Figures 4A and 4B). It is impossible to directly measure inner mitochondrial membrane channel activity in intact cells because the inner membrane is inaccessible to patch pipettes. Thus, we relied on an imaging method that allows measurement of changes in mitochondrial membrane potential. As reported by He et al. (2017b), when the calcium ionophore ferutinin is added to c-subunit KO cells it induces CSA-sensitive mitochondrial membrane depolarization (Abramov and Duchen, 2003). This depolarization can be monitored in intact cells using the fluorescent probe TMRM. TMRM is a positively charged fluorescent probe that accumulates inside polarized mitochondria and leaks out when the membrane becomes depolarized. Notably, if TMRM leak is inhibited this would indicate the inhibition of both low and high conductance channels. Figure 4C shows representative images of TMRM fluorescence in c-subunit KO cells before and after addition of 10 μM of ferutinin. Both CSA (4 μM) and BA (5 μM) significantly (p < 0.01, n = 4) delayed the activation of mPTP which resulted in a prolonged delay between the addition of ferutinin and the membrane depolarization (Figures 4E and 4F). This indicates that in KO cells similarly to CSA, BA can inhibit calcium induced mitochondrial depolarization.

In the wild-type HAP1 cells containing c-subunit under identical experimental conditions CSA strongly inhibited calcium induced depolarization (Figure 4D). Interestingly in these cells mPTP activation required a longer time and was more strongly inhibited by CSA compared to c-subunit KO cells (46 ± 10 s, n = 6 for the KO vs. 210 ± 22, n = 6 for wild-type [WT], p < 0.001) (Figure 4F). In fact, in the WT cells in the presence of the same amounts of ferutinin, CSA completely blocked mPTP and only mild depolarization was detected over the time of experiment (Figure 4D, blue trace). However, BA did not produce significant inhibition compared to control (Figure 4F), suggesting that part of the CSA-sensitive depolarizing component is missing or attenuated. Notably, in the WT cells in the presence of CSA depolarization never reached 50% in the time frame of our experiment and thus CSA bar graph is not present. This suggests that not only channel conductance but also the regulation of the calcium-induced membrane depolarization is significantly altered in c-subunit KO HAP1-A12 cells compared to the HAP1 cells.

## DISCUSSION

In this study we demonstrate that in the absence of ATP synthase c-subunit, CSA-sensitive channel activity is distinctly different from the activity of the mPTP channel of WT mitochondria. This suggests that the c-subunit plays a critical role in formation of the native calcium-induced mPTP channel.

We consider two likely possibilities that can explain this phenomenon. First, it is possible that in the absence of ATP synthase c-subunit channel conductance of the “bona fide” mPTP is reduced. This scenario would likely take place in a model that does not require formation of the pore by oligomers of the c-subunit (Bernardi et al., 2015; Carraro et al., 2014). In this scenario, absence of the c-subunit might be hypothesized to disrupt the native conformation of the complex and by doing so could modify the single channel behavior of mPTP. Interestingly, a very recent study in yeast suggests that deletions in other parts of the ATP synthase leads to the appearance of small conductance channels at the level of purified complex (Bernardi, 2018; Carraro et al., 2018). Another possibility is that in the absence of the native mPTP, channel membrane permeabilization linked to PT can be achieved by a complimentary mechanism possibly through activation of the ANT pore. We favor the second possibility because the size of the purified reconstituted ANT pore is significantly smaller than that of the native mPTP (Brustovetsky and Klingenberg, 1996; Kinnally et al., 1996) and is comparable in size to the channel seen in our experiments where mitoplasts lack c-subunit. Further, recombinant ANT forms a pore in the presence of elevated concentrations of calcium and this pore can become CSA dependent upon addition of CypD (Brustovetsky and Klingenberg, 1996). It has also been demonstrated that in intact mitochondria ANT interacts with CypD (Halestrap and Davidson, 1990). In our experiments this ANT-like activity recorded in the absence of c-subunit was sensitive to CSA in addition to BA and ADP. Further, a very recent study of triple ANT KO cells shows that ANT still could be involved in PTP (Karch et al., 2018). However, in this study of triple ANT KO, there continues to be exquisite CSA sensitivity, suggesting the CSA regulation of mPTP is still present in the ANT KOs. Future patch-clamp experiments will likely help to clarify this question. Altogether our findings suggest that mPTP formation can potentially occur by (at least) two CypD dependent pathways, one involving ATP synthase and the other involving ANT. If this is the case, then the primary mechanism of mPTP would likely involve the larger pore, that is, assembly of the c-subunit ion conducting complex (Elustondoet al., 2016), whereas a secondary mechanism of pore formation would involve participation of ANT, but perhaps only in the absence of c-subunit. It is also conceivable that absence of c-subunit causes changes in the conformation of the native mPTP that lead to lower conductance.

CypD is a chaperone foldase that is potentially involved in interaction with multiple membrane proteins. It was earlier proposed that during oxidative stress mPTP develops through CypD interaction with unfolded proteins (Lemasters et al., 1998). It is also noteworthy that SPG7 peptidase that has been recently proposed to play a critical role in PT development is an enzyme that is also involved in protein processing and folding (Shanmughapriya et al., 2015). CypD-protein interactions would explain why many CSA-sensitive channels can be found even in genetically modified mitochondria designed to eliminate pore candidates. Under this scenario during conditions of pathological calcium elevation, mPTP may occur through (mis-)folding of multiple membrane proteins to induce channel-conducting conformations. Single or multiple KO strategies and further investigations will be required to test this possibility.

It has been estimated in experiments using intact mitochondria that the physical size of the mPTP is expected to be in the range of 2–3 nm (discussed by Zoratti and Szabò [1995]). Therefore, it can be estimated that a water-filled 2- to 3-nm pore in 150 mM KCl solution is expected to have an average conductance in the range of 0.7 nS to 1.6 nS (Pavlov et al., 2001). These values correspond well with the patch-clamp measurements of mPTP in the WT mitochondria. However, the lower conductance of the CSA-sensitive channel in c-subunit KO mitochondria suggests that the physical size of this channel is smaller than expected for WT mPTP. In addition, the decreased CSA sensitivity of mitochondrial membrane depolarization upon ferutinin exposure suggests that the total CSA-sensitive conductance is substantially decreased in c-subunit depleted cells.

Interestingly, two cells types not only differed in CSA-sensitive channel activities detected by patch-clamp but also in sensitivity to mPTP induction at the level of the intact cells. In agreement with the previous report by He et al. (2017b), in both cell types ferutinin induced CSA-sensitive mitochondrial membrane depolarization. However, we found that KO cells more easily undergo mPTP compared to the WT cells but that WT cell are stronger inhibited by CSA, suggesting that proper operation and structure of the ATP synthase contributes both to protecting from membrane potential loss and to the marked sensitivity to CSA. Further, unlike in KOHAP1 – A12 cells, mPTP in WT HAP1 cells was not sensitive to BA. These results allow the possibility that in KO cells PT occurs by a mechanism that is distinct from WT cells containing c-subunit.

In summary, we found that in c-subunit-deficient mitochondria CSA-sensitive channel activity is distinctly different from the mPTP channel of WT mitochondria. We suggest that, although the c-subunit is likely to be the primary contributor to the activity of the WT mPTP, Ca^2+^-induced mitochondrial membrane depolarization can potentially be caused by alternative channels with a common mechanism of CypD sensitivity. Alternatively, the absence of the c-subunit might enhance mPTP formation that involves either ANT or other proteins.

## STAR★METHODS

### CONTACT FOR REAGENT AND RESOURCE SHARING

Further information and requests for resources and reagents should be directed to and will be fulfilled by the Lead Contact, Evgeny Pavlov (ep37@nyu.edu).

### EXPERIMENTAL MODEL AND SUBJECT DETAILS

Cultured HAP1 cells containing c–subunit and HAP1 – A12 cells that are lacking C subunit of ATP synthase were used for the study. The cells were cultured as described previously (He et al., 2017a). Both cell types were grown on Iscove’s Modified Dulbecco’s Medium (IMDM), supplemented with 10% FBS and 20 units/mL Penicillin/Streptomycin and maintained in a humidified cell incubator, at 37°C under a 5% CO_2_ atmosphere. Cells were plated on Petri dishes and, 24h later, used for mitoplast preparation.

### METHOD DETAILS

#### Mitoplast preparation

Mitochondria from HAP1 and HAP1 – A12 cells were isolated by homogenization and differential centrifugation in mannitol-sucrose buffer (225 mM mannitol, 75 mM sucrose, 5 mM Tris-HCl, pH = 7.4) completed by 1 mM of PMSF (Thermo Fisher), EDTA-free protease inhibitor cocktail (Sigma Aldrich) and 1 mM of EGTA (Sigma Aldrich). Briefly, cells were washed 2-3 times on cold PBS and scraped on the mannitol-sucrose buffer. After that, they were collected on a glass tube and grinded 80 times with a pestle, always on ice. Cells were then centrifuged at 600 xg for 5 min at 4°C and supernatants were collected and centrifuged again, using the same conditions. After that, supernatants were collected and centrifuged once more at 4°C for 5 min, but using this time 10.300 xg. Then, pellets were resuspended in 100 μL of the mannitol-sucrose buffer and centrifuged at 10.300 xg, for 10 min at 4°C. Supernatants were discarded and isolated mitochondria were incubated in isotonic KCl solution (150 mM KCl; 5 mM HEPES, pH 7.4) containing 4 mM of succinate, 2 μM of rotenone and 2 mM of CaCl_2_ for 10-15 min that leads to swelling of the mitochondria and rupture of the mitochondrial outer membrane. Mitoplast containing solution was placed to glass bottom chamber for several minutes to allow mitoplast to settle to the bottom of the chamber, after that attached mitoplasts were gently washed 2 times by KCl solution.

#### Patch-clamp study

Patch-clamp procedures and analysis used were described previously (Kinnally et al., 1991; Pavlov et al. 2001). Briefly, membrane patches were excised from mitoplasts after formation of a giga-seal using micropipettes with resistances of 20–40 MΩ at room temperature. Solution for patch-clamp recordings was symmetrical 150 mM KCl, 5 mM HEPES, pH 7.4. Voltage clamp was performed with the excised configuration of the patch-clamp technique using one channel amplifier eONE (Elements, Cesena, Italy) in the insideout mode. Voltages are reported as pipette potentials. Cyclosporine A (CSA, Sigma Aldrich), bongkrekic acid (BA, Abcam) and ADP (Sigma Aldrich) were used for inhibition of channel activity.

#### Imaging

For imaging experiments HAP1 and HAP1 – A12 cells were incubated with 10 nM TMRM for 30 minutes in a HEPES-buffered salt solution (HBSS) composed of (mM): 156 NaCl, 3 KCl, 2 MgSO_4_, 1.25 KH_2_PO_4_, 2 CaCl_2_, 10 glucose and 10 HEPES; pH adjusted to 7.35 with NaOH and then the different reagents were added at different concentrations. In confluency of the cells were kept at approximately 50%. Images were obtained using a Nikon (Chiyoda, Tokyo, Japan) fluorescent microscope with LED light source (Crestoptics S.p.A.) and Andor CCD camera. The LED light source power was kept at minimal values in order to avoid phototoxicity. For time lapse experiments cells were imaged every 5 s at a 20x or 40x magnification using air objective. Average fluorescence intensity from 10 ROIs from mitochondrial regions was used for data analysis to obtain average times of depolarization. These data were from combined from 3 to 6 independent experiments for comparison between average values of different treatment conditions.

#### Western blotting

Mitochondria isolated from HAP1 and HAP1 – A12 cells were screened for the presence of the c-subunit by western blotting. The antibody against all three isoforms of c-subunit was used to detect c-subunit levels (ab 180149, Abcam). The antibody against ANT1 was used to measure the endogenous level of slc25a4 protein in HAP1 and HAP1-A12 cells (32484, Sabbiotech). VDAC1 was used as a protein loading control.

### QUANTIFICATION AND STATISTICAL ANALYSIS

Clampfit 10.7 (Molecular Devices, CA, USA) and Origin 2018 (OriginLab. Corporation, Northampton, Massachusetts, USA) were used for analysis of channel activity and statistical analysis. Student’s t test was used to determine significant differences in the conductance values. The data are represented as mean ± SEM. The number of current recordings (n) for each type of experiment is outlined in the figure legends.

NIS-Elements AR 4.30.02 software was used to analyze images. Average fluorescence intensity from 10 ROIs from mitochondrial regions was used for data analysis to obtain average times of depolarization. These data were from combined from 3 to 6 independent experiments for comparison between average values of different treatment conditions. Student’s t test was used to determine significant differences in the time of 50% depolarization.

## Supplementary Material

1

2

## Figures and Tables

**Table:** KEY RESOURCES TABLE

REAGENT or RESOURCE	SOURCE	IDENTIFIER
Antibodies		
Rabbit monoclonal [EPR13908] to ATP5G1/G2/G3	Abcam	Cat # ab180149, RIID: AB_180149
Rabbit polyclonal to SLC25A4	Sabbiotech	Cat # 32484, RIID: AB_32484
Rabbit polyclonal to VDAC1 / Porin - Mitochondrial Loading Control	Abcam	Cat# ab15895, RRID: AB_2214787
Chemicals, Peptides, and Recombinant Proteins		
Cyclosporine A	Sigma Aldrich	S 7481F1; CAS: 59865-13-3
Bongkrekic acid	Abcam	ab142111; CAS: 11076-19-0
ADP	Sigma Aldrich	A2754; CAS: 20398-34-9
TMRM	Invitrogen	T668
PMSF	Thermo Fisher	36978; CAS: 329-98-6
Critical Commercial Assays		
EDTA-free protease inhibitor cocktail	Sigma Aldrich	Cat# 11836170001 Roche
Experimental Models: Cell Lines		
HAP1 cells	He etal., 2017a	N/A
HAP1-A12 cells	He etal., 2017a	N/A
Software and Algorithms		
Clampfit 10.7	Molecular Devices, CA, USA	N/A
Origin 2018	OriginLab, Massachusetts, USA	N/A
Other		
Channel amplifier eONE for patch-clamp	Elements, Cesena, Italy	N/A

## Highlights

- Deletion of the c-subunit leads to loss of the mPTP channel
- C-subunit KO mitochondria contain a CSA-sensitive channel
- The c-subunit KO channel has lower conductance compared to mPTP
- The c-subunit KO channel is sensitive to ANT inhibitors
