# Supplementary material for: ATP Synthase C-Subunit-Deficient Mitochondria Have a Small Cyclosporine A-Sensitive Channel, but Lack the Permeability Transition Pore
Source: Cell Rep. Author manuscript; Available in PMC 2019 May 16. (PMC6521848; doi:10.1016/j.celrep.2018.12.033)
Supplement: 1 [file NIHMS1517817-supplement-1.pdf]

Cell Reports, Volume *26*

Supplemental Information

ATP Synthase C-Subunit-Deficient Mitochondria

Have a Small Cyclosporine A-Sensitive Channel,

but Lack the Permeability Transition Pore

Maria A. Neginskaya, Maria E. Solesio, Elena V. Berezhnaya, Giuseppe F. Amodeo, Nelli Mnatsakanyan, Elizabeth A. Jonas, and Evgeny V. Pavlov

Figure S1

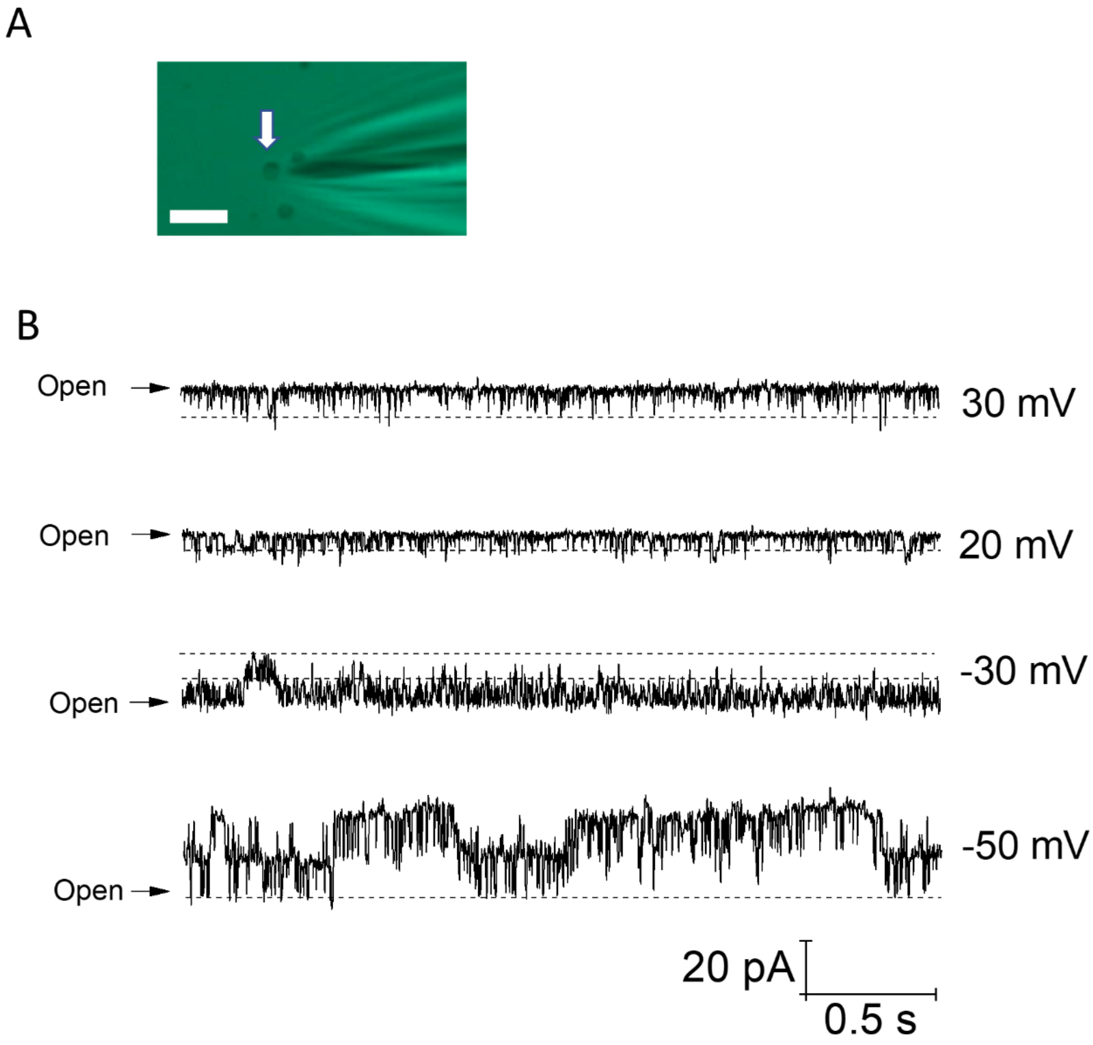

Figure S1. Related to Figure 2D. TIM channel activity in c-subunit KO mitochondria. A) Phase contrast image of the mitoplast and patch-clamp pipette. Note the dark "cap" on the top of the mitoplast formed by the remains of the mitochondrial outer membrane. Scale bar: 5 microns; B) Voltage dependent gating of the CSA insensitive channel. Note tendency toward increased flickering and transition to the substate at the negative voltage – properties previously described for Translocator of the Inner Membrane (TIM) channel.
